# Prognostic significance of the co-expression of EGFR and HER2 in adenocarcinoma of the uterine cervix

**DOI:** 10.1371/journal.pone.0184123

**Published:** 2017-08-31

**Authors:** Asako Ueda, Akira Takasawa, Taishi Akimoto, Kumi Takasawa, Tomoyuki Aoyama, Yoshihiko Ino, Masanori Nojima, Yusuke Ono, Masaki Murata, Makoto Osanai, Tadashi Hasegawa, Tsuyoshi Saito, Norimasa Sawada

**Affiliations:** 1 Department of Pathology, Sapporo Medical University School of Medicine, Sapporo, Japan; 2 Department of Obstetrics and Gynecology, Sapporo Medical University School of Medicine, Sapporo, Japan; 3 Department of Surgical Pathology, Sapporo Medical University School of Medicine, Sapporo, Japan; 4 Division of Advanced Medicine Promotion, The Advanced Clinical Research Center, The Institute of Medical Science, The University of Tokyo, Tokyo, Japan; National Cancer Center, JAPAN

## Abstract

Prognostic factors and therapeutic targets are needed for the patients with cervical adenocarcinoma because they have a poor prognosis. Recently, co-expression of multiple receptor tyrosine kinases (RTKs) has been found to be associated with aggressive biological behavior and poor prognosis of several types of malignancy. To evaluate the significance of the expression of multiple RTKs in uterine cervical cancers, we examined the expression profile of RTKs (EGFR, HER2 and c-Met) and the correlation of their expression with clinicopathological features and prognosis of patients with cervical adenocarcinomas. AIS and adenocarcinoma showed strong expression of a single RTK (EGFR, HER2 or c-Met) on the cell membrane in 41 (77.4%) of 53 cases. Twenty (46%) of the 43 adenocarcinoma cases were positive for double or triple RTKs (P = 0.034). Positivity for EGFR and double positivity for EGFR and HER2 (EGFR+/HER2+/c-Met+ and EGFR+/HER2+/c-Met-) were significantly correlated with lymph node metastasis (P = 0.010 for single and P = 0.013 for double) and UICC stage (P = 0.021 for single and P = 0.007 for double). Positivity for HER2 was significantly correlated with tumor size (P = 0.029). Relapse-free survival (RFS) was significantly shorter in patients who were double positive for EGFR and HER2. Our results suggest that EGFR and HER2 are potential therapeutic targets and that their co-expression is a prognostic factor for cervical adenocarcinoma.

## Introduction

The incidence of cervical carcinoma has remained very high worldwide, especially in developing countries [1]. Interestingly, in developed countries including Japan, the incidence of cervical squamous cell carcinoma has been decreasing, whereas the incidence of cervical adenocarcinoma in young women has significantly increased [1–4]. In addition, patients with cervical adenocarcinoma have a worse prognosis, with earlier local extension, lymph node metastasis and chemoradiation resistance, than do patients with squamous cell carcinoma of the cervix [5–7]. To improve the prognosis, new useful diagnostic markers and therapeutic targets are required for adenocarcinoma [8–10].

Recently, the expression of receptor tyrosine kinases (RTKs), EGFR, HER2 and c-Met, has been considered in connection with uterine cervix adenocarcinoma. RTKs are growth factor receptors with tyrosine kinase activity that are present on the cell membrane [11,12]. The percentage of cervical adenocarcinoma cases in which EGFR expression was detected varied from 19% to 67% in previous studies, and its overexpression was shown to be associated with poor prognosis [8,13–17]. In contrast, other investigators found that EGFR was not an indicator of prognosis for patients with cervical cancer including adenocarcinoma [14,18,19]. It was reported that HER2 was overexpressed in 24%-49% of cervical adenocarcinoma cases and that its overexpression was associated with more advanced disease stage and worse prognosis [15,20,21]. It was shown that c-Met was overexpressed in 30%-67% of cervical squamous cell carcinoma cases, and one study showed that c-Met expression was present in 30% of cervical adenocarcinoma cases [22–24]. Overexpression of c-Met was shown to be associated with more advanced stage and poor prognosis [25].

RTK signaling pathways play essential roles in cell proliferation, differentiation, survival, migration, and adhesion. Therefore, the presence of RTKs has been shown to be associated with aggressive biological behavior, poor prognosis and therapeutic resistance for several types of malignancy [26–28] including cervical squamous cell carcinoma [29–33]. Furthermore, co-overexpression of EGFR and HER2 has been reported to be associated with malignancy in several tumors including breast cancer, lung cancer, prostate cancer and urinary bladder cancer [34,35]. Those studies suggest that oncogenic transformation might be accelerated by co-expression of multiple RTKs [15,36]. Although these RTKs are known to play a key role in oncogenic transformation, carcinogenesis and tumor invasiveness, there is little information about the relationships between EGFR, HER2 and c-Met in cervical adenocarcinoma.

In this study, we examined the expression of receptor tyrosine kinases, EGFR, HER2 and c-MET, in cervical adenocarcinomas to determine whether they are useful as prognostic factors and therapeutic targets of cervical cancers, with focus on the importance of co-expression of multiple RTKs.

## Materials and methods

### Patients and specimens

Specimens of 53 cases of cervical adenocarcinoma including adenocarcinoma *in situ* (AIS) obtained by surgical resections during the period from 2004 to 2012 were retrieved from the pathology file of Sapporo Medical University Hospital, Sapporo, Japan. The protocol for human study was reviewed and approved by the ethics committee of Sapporo Medical University School of Medicine. Written informed consent was obtained from each patient who participated in the investigation. The mean age of the patients was 43 years (range, 25–79 years). The histological type was based on WHO classification of tumors of the uterine cervix (4th edition) [1]. The histological diagnosis included endocervical type “MuE”(n = 33, 62.2%), intestinal type “MuI” (n = 4, 7.5%), minimal deviation type “MuM” (n = 3, 5.7%), villoglandular type “MuV” (n = 3, 5.7%) and adenocarcinoma *in situ* “AIS” (n = 10, 18.9%). The 53 cases were staged by the Union for International Cancer Control (Unio Internationalis Contra Cancrum, UICC) stage classification (7th edition): stage 0 (n = 10), stage IA (n = 6), stage IB (n = 26), stage IIA (n = 4), stage IIB (n = 1), and stage IIIB (n = 6).

### Clinicopathological data

We retrospectively collected clinicopathological data for age, histological type, tumor size, lymph node metastasis, lymphovascular infiltration, UICC stage, relapse-free survival (RFS) and overall survival (OS).

### Immunohistochemical staining of surgical specimens

Hematoxylin and eosin (H&E)-stained slides from all cases were reviewed to select representative sections. New sections were prepared from paraffin blocks of formalin-fixed surgical specimens and were immunohistochemically stained. Sections were dewaxed, rehydrated, and moistened with phosphate-buffered saline (PBS) (pH 7.4). Antigen retrieval was performed by Proteinase K treatment (EGFR), heat-induced epitope retrieval by using the Benchmark XT system (HER2) and by using a microwave in Tris-EDTA (pH 9.0) for 30 min (c-Met). Primary antibodies were EGFR (31G7, Nichirei, x100), HER2/ner (4B5, Roche, x100) and c-Met (D1C2, Cell Signaling, x100). Secondary antibody and detection was Dako Real^TM^ EnVision^TM^ detection system (EGFR and c-Met) and Ventana iVIEW DAB detection kit (HER2) [37].

### Immunohistochemical analysis

Evaluation of immunoreactivity was based on a semiquantitative analysis, manually scored as the percentage of positive cells. Only epithelial cells with membrane staining were included in the analysis. Surgical specimen staining patterns were scored as follows: score 0, no reactivity or membranous reactivity in less than 10% of tumor cells: score 1+, faint/almost no membranous reactivity in 10% or more of tumor cells; score 2+, weak to moderate complete or basolateral membranous reactivity in 10% or more tumor cells; and score 3+, moderate to strong complete or basolateral membranous reactivity in 10% or more of tumor cells. For statistical purposes, samples with scores (0) and (1+) were considered negative, and those with scores (2+) and (3+) were considered positive. When evaluating the slides, the observers (A. T, A. U, T. A) were blinded to the clinical data. Discordant cases were discussed, and a consensus was reached.

### Statistical analysis

Statistical analysis was performed using Pearson’s χ^2^ test, Fisher’s exact test, the Kruskal-Wallis test, and the logrank test. Kaplan-Meier curves were generated for the positive group (immunoreactivity of 2+ to 3+) and the negative group (immunoreactivity of 0 to 1+) for either single, double or triple RTKs. All statistical analyses were performed with EZR (Saitama Medical Center, Jichi Medical University, Saitama, Japan) [38], which is a graphical user interface for R (The R Foundation for Statistical Computing, Vienna, Austria). More precisely, it is a modified version of R commander designed to add statistical functions frequently used in biostatistics.

## Results

### Clinical and pathological findings

Patient characteristics and clinicopathological characteristics are summarized in Table 1. The study population consisted of 53 patients with an age range of 25 to 79 years. The median age of the patients was 43 years. The clinical stage status of the patients was determined by UICC classification (0 = 10, IA = 5, IB = 27, IIA = 4, IIB = 1, and IIIB = 6). Endocervical type adenocarcinoma (MuE) was the most common histological type. The frequencies of lymph node metastasis and lymphovascular infiltration were 11.3% and 28.3%, respectively.

**Table 1:** Clinicopathological features of cervical adenocarcinomas.

Patients (n = 53)	
Age (range, median)	25–79, 43
Histological type	
Adenocarcinoma	43
Endocervical type (MuE)	33
Intestinal type (MuI)	4
Minimal deviation type (MuM)	3
Villoglandular type (MuV)	3
AIS (Adenocarcinoma in situ)	10
Tumor stage (UICC)	
0	10
IA	5
IB	27
IIA	4
IIB	1
IIIA	0
IIIB	6
Tumor size	
AIS only	10
< = 40mm	31
>40mm	12
Lymph node metastasis	
Negative	47
Positive	6
Lymphovascular infiltration	
Negative	38
Positive	15

### Immunohistochemistry of receptor tyrosine kinases (RTKs) in cervical adenocarcinoma

**Fig 1:**
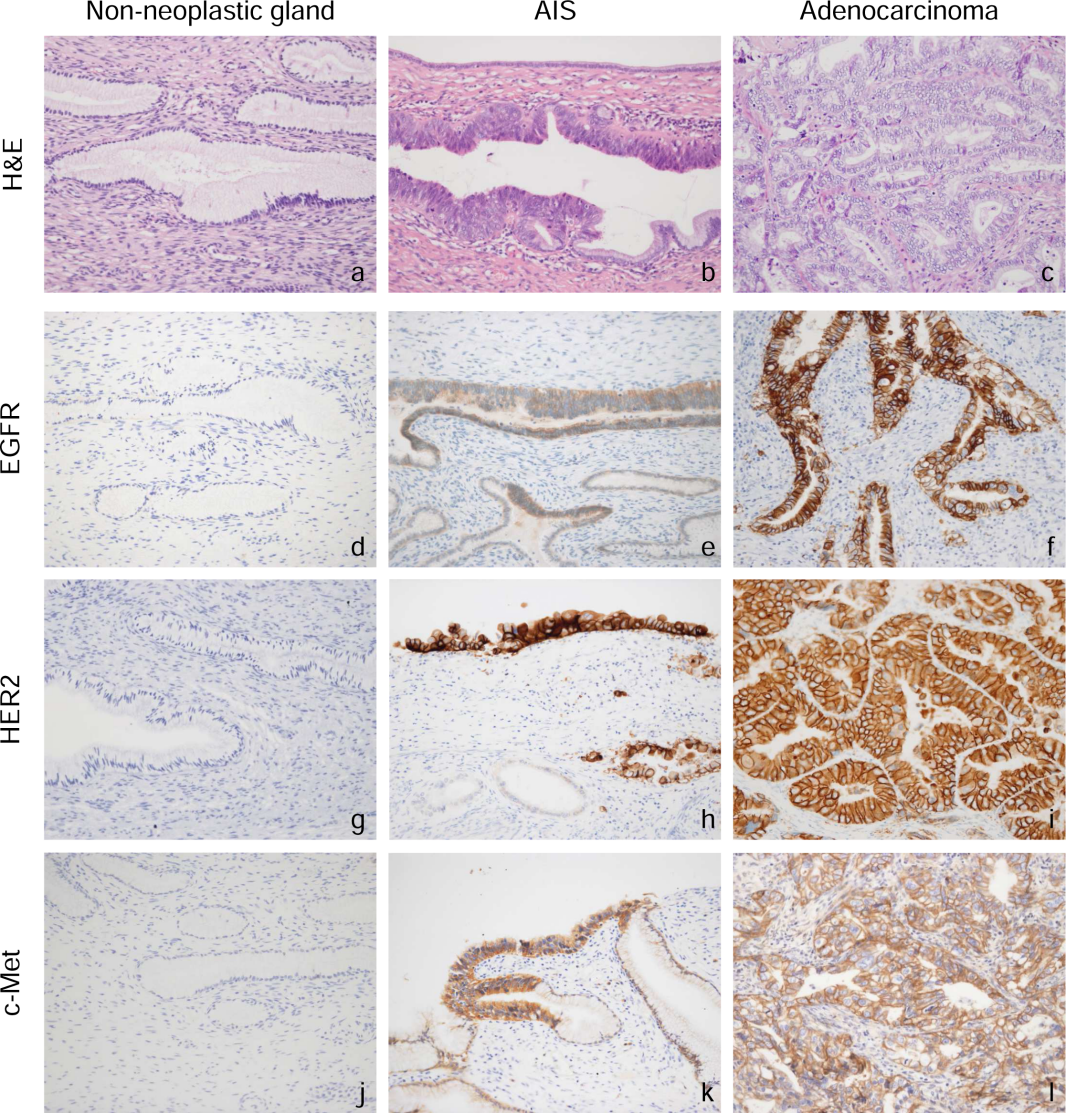
H&E staining and immunohistochemical staining in surgical specimens of non-neoplastic cervical glands, adenocarcinoma in situ (AIS), and cervical adenocarcinoma. Representative immunohistochemical staining of EGFR (d-f), HER2 (g-i), and c-Met (j–l) is shown. The receptor tyrosine kinases were predominantly expressed on the membranes of tumor cells.

Immunohistochemistry was performed on surgical specimens by using antibodies for EGFR, HER2 and c-Met. In non-neoplastic cervical gland tissue, expression of RTKs (EGFR, HER2 and c-Met) was faint or absent (Fig 1). In contrast, adenocarcinoma *in situ* (AIS) and adenocarcinoma showed strong expression of RTKs (EGFR, HER2 or c-Met) on the cell membrane in 41 (77.4%) of the 53 cases. Among the three receptors, c-Met was the most frequently detected, being positive in 28 (52.8%) of the 53 cases, including 13 cases (24.5%) with an immunoreactive score (IRS) of 3+ and 15 cases (28.3%) with IRS of 2+ (Table 2). HER2 was positive in 24 (45.3%) of the 53 cases, including 10 cases (18.9%) and 14 cases (26.4%) with IRS of 3+ and 2+, respectively. EGFR was positive in 17 (32.1%) of the 53 cases, including 7 cases (13.2%) and 10 cases (18.9%) with IRS of 3+ and 2+, respectively.

**Table 2:** Immunoreactive intensity of RTKs in cervical adenocarcinomas.

	EGFR				HER2				c-Met			
Intensity	0	1+	2+	3+	0	1+	2+	3+	0	1+	2+	3+
AIS	6	3	1	0	5	2	2	1	4	2	2	2
Adenocarcinoma	20	7	9	7	11	11	12	9	10	9	13	11

**Fig 2:**
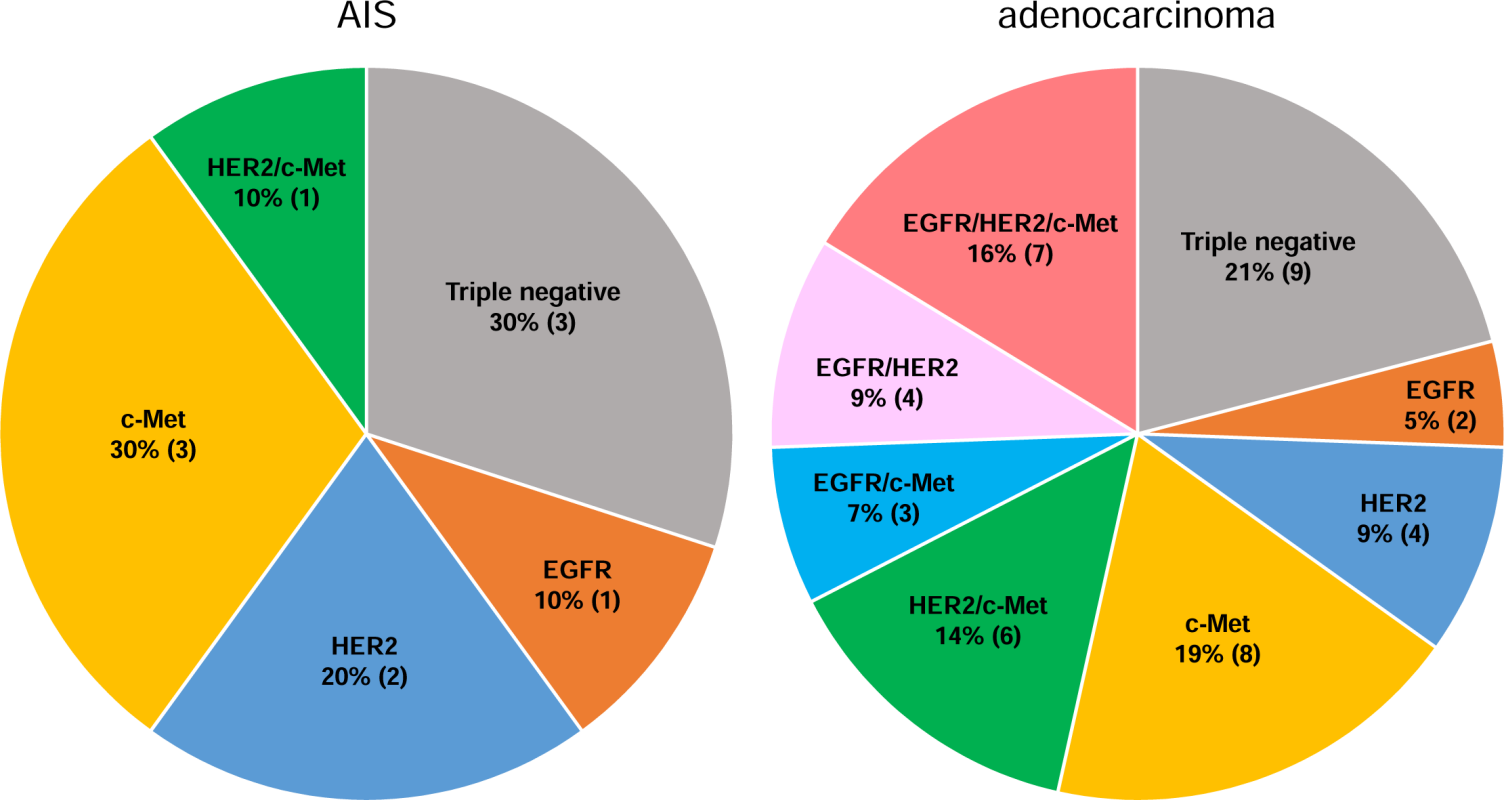
Expression profiles of receptor tyrosine kinases in cervical adenocarcinoma. One (10%) of the 10 AIS cases exhibited a multiple simultaneous positive status. In adenocarcinoma, 20 cases (46%, n = 43) exhibited multiple simultaneous positive status. The percentage of cases with positive expression of multiple RTKs was significantly higher in adenocarcinoma than in AIS (Fig 2, chi-square test, p = 0.034).

The percentage of cases with positive expression of multiple RTKs was significantly higher in adenocarcinoma than in AIS (Fig 2, chi-square test, p = 0.034). Twenty (46%) of the 43 adenocarcinoma cases were positive for multiple RTKs, including 7 cases (16%) with EGFR+/HER2+/c-Met+, 4 cases (9%) with EGFR+/HER2+/c-Met-, 3 cases (7%) with EGFR+/HER2-/c-Met+, and 6 cases (14%) with EGFR-/HER2+/c-Met+. Eleven adenocarcinoma cases (25%) were double positive for EGFR and HER2 (EGFR+/HER2+/c-Met+ and EGFR+/HER2+/c-Met-) (Fig 2). In AIS, only one case (10%) was double positive for HER2 and c-Met.

### Positive correlation between expression of receptor tyrosine kinases (RTKs) and clinicopathological features of cervical adenocarcinoma

As shown in Table 3, EGFR positivity was significantly correlated with lymph node metastasis (P = 0.010) and UICC stage (P = 0.021); however, there was no significant correlation between EGFR positivity and patient’s age (P = 0.387), histological type (P = 0.14) or lymphovascular infiltration (P = 0.197). HER2 positivity was correlated only with tumor size (P = 0.0291). c-Met positivity had no correlation with any clinicopathological features.

**Table 3:** Analyses of correlation between expression of a single RTK and clinicopathological features.

		EGFR			HER2			c-Met		
	N	0–1+	2+-3+	P value	0–1+	2+-3+	P value	0–1+	2+-3+	P value
Age										
Age under median (< = 43)	27	20 (74.1%)	7 (25.9%)	0.387	15 (55.6%)	12 (44.4%)	1	12 (44.4%)	15 (55.6%)	0.786
Age over median (>43)	26	16 (61.5%)	10 (38.5%)		14 (46.2%)	12 (53.8%)		13 (50%)	13 (50%)	
Histological type										
AIS	10	9 (90%)	1 (10%)	0.140	7 (70%)	3 (30%)	0.318	6 (60%)	4 (40%)	0.488
Adenocarcinoma	43	27 (62.8%)	16 (37.2%)		22 (51.2%)	21 (48.8%)		19 (44.2%)	24 (55.8%)	
Tumor size										
AIS	10	9 (90%)	1 (10%)	0.151	7 (70%)	3 (30%)	0.029	6 (60%)	4 (40%)	0.702
< = 40mm	31	21 (67.7%)	10 (32.3%)		19 (61.3%)	12 (38.7%)		13 (41.9%)	18 (58.1%)	
>40mm	12	6 (50%)	6 (50%)		3 (25%)	9 (75%)		6 (50%)	6 (50%)	
Lymph node metastasis										
Negative	47	35 (74.5%)	12 (25.5%)	0.010	27 (57.4%)	20 (42.6%)	0.392	23 (48.9%)	24 (51.1%)	0.672
Positive	6	1 (16.7%)	5 (83.3%)		2 (33.3%)	4 (66.7%)		2 (33.3%)	4 (66.7%)	
Lymphovascular infiltration										
Negative	38	28 (73.7%)	10 (26.3%)	0.197	21 (55.3%)	17 (44.7%)	1	18 (47.4%)	20 (52.6%)	1
Positive	15	8 (53.3%)	7 (46.7%)		8 (53.3%)	7 (46.7%)		7 (46.7%)	8 (53.3%)	
UICC stage										
0	10	9 (90%)	1 (10%)	0.021	7 (70%)	3 (30%)	0.463	6 (60%)	4 (40%)	0.478
I	32	22 (68.8%)	10 (31.2%)		18 (56.3%)	14 (43.7%)		14 (43.7%)	18 (56.3%)	
II	5	4 (80%)	1 (20%)		2 (40%)	3 (60%)		3 (60%)	2 (40%)	
III	6	1 (16.7%)	5 (83.3%)		2 (33.3%)	4 (66.7%)		2 (33.3%)	4 (66.7%)	

Regarding positivity of multiple RTKs, double positivity for EGFR and HER2 (EGFR+/HER2+/c-Met+ and EGFR+/HER2+/c-Met-) was significantly correlated with tumor size in addition to lymph node metastasis and UICC stage, which were also correlated with single EGFR positivity (Table 4). Other combinations of RTK positivity showed no correlation with any clinicopathological features.

**Table 4:** Analyses of correlation between expression of multiple RTKs and clinicopathological features.

		EGFR + HER2		
	N	0–1+	2+-3+	P value
Tumor size				
AIS	10	10 (100%)	0 (0%)	0.017
< = 40mm	31	25 (80.6%)	6 (19.4%)	
>40mm	12	7 (58.3%)	5 (41.7%)	
Lymph node metastasis				
Negative	47	40 (85.1%)	7 (14.9%)	0.013
Positive	6	2 (33.3%)	4 (66.7%)	
UICC stage				
0	10	10 (100%)	0 (0%)	0.007
I	32	26 (81.3%)	6 (18.7%)	
II	5	4 (80%)	1 (20%)	
III	6	2 (33.3%)	4 (66.7%)	

### Positive correlation between expression of multiple receptor tyrosine kinases (RTKs) and survival of patients with cervical adenocarcinoma

**Fig 3:**
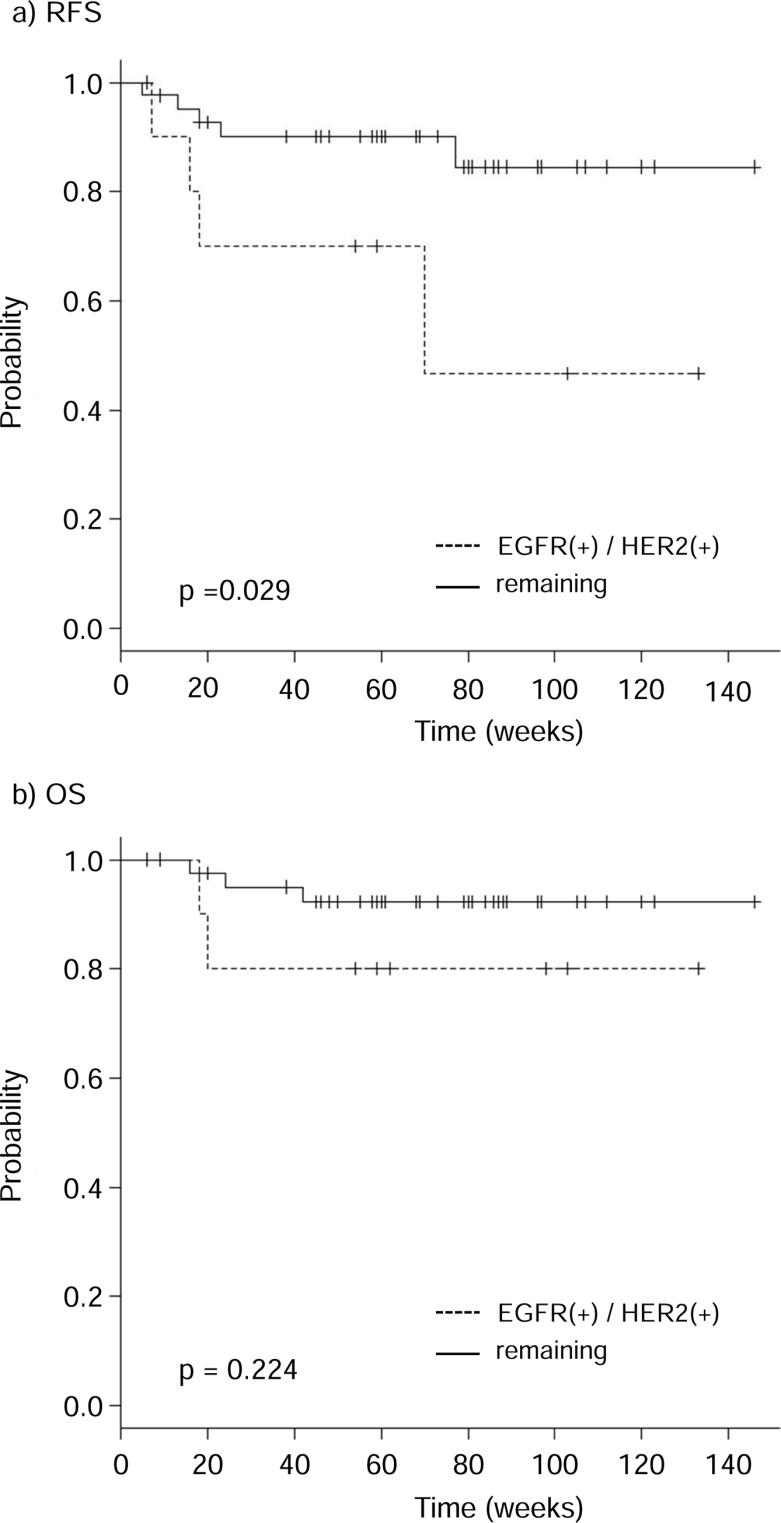
Kaplan-Meier estimates of relapse-free survival (RFS) and overall survival (OS) of patients with cervical adenocarcinoma according to the combined expression of EGFR and HER2. The patients were classified into two groups: those with high expression levels of EGFR and HER2 (dashed lines) and the remaining patients (solid lines). High expression levels of both EGFR and HER2 were significantly correlated with worse RFS (p = 0.029).

The relationships of the expression of RTKs in cervical adenocarcinoma with relapse-free survival (RFS) and overall survival (OS) were assessed by using the Kaplan-Meier method. There was no correlation between single RTK positivity and RFS or OS (S1 Fig). Regarding positivity of multiple RTKs, RFS was significantly shorter in patients who were double positive for EGFR and HER2 (EGFR+/HER2+/c-Met+ and EGFR+/HER2+/c-Met-) than in the remaining patients (Fig 3). The association between RFS and other combinations of double positive cases (EGFR+/c-Met+ and HER2+/c-Met+) was not significant (S2 Fig). There was no correlation between overall survival (OS) and any combination of RTK positivity. Multivariate analysis would have been helpful for evaluation here, but it was difficult to perform properly due to an insufficient number of events (< 10).

## Discussion

This is the first study to evaluate the impact of co-expression of RTKs (EGFR, HER2, and c-Met) on relapse-free survival and overall survival of patients with cervical adenocarcinoma.

The presence of RTKs has been reported to be associated with accelerated tumor progression and therapeutic resistance for several types of malignancies, including cervical cancer [26–33]. However, most of the reports on cervical cancers have examined cervical ‘squamous cell carcinoma (SCC)’, as in the report by Shen et al. whereas there are only a few reports on cervical ‘adenocarcinoma’ including our current study [32]. Pathologically, SCC and adenocarcinoma are quite different. Patients with cervical adenocarcinoma have a worse prognosis, with earlier local extension, lymph node metastasis and chemoradiation resistance, than do patients with squamous cell carcinoma of the cervix [5–7]. Therefore, SCC and adenocarcinomas should be discussed separately. It is very important to examine the expression profile of RTKs with a focus on cervical adenocarcinoma across races, countries and centers because several RTK inhibitors have been already available for clinical practice of other malignancies.

In this study, more than 30% of adenocarcinoma and AIS cases showed strong staining for EGFR, whereas non-neoplastic cervical glands showed no staining or only faint staining. EGFR positivity was associated with lymph node metastasis and UICC stage of the patients, which may result from the well-known role of EGFR in proliferation, invasiveness and migration of tumor cells. However, there was no correlation between EGFR positivity and prognosis of the patients, as some groups have reported [14,18,19]. In contrast, Pérez-Regadera et al. reported that overexpression of EGFR was significantly associated with decreased disease-free survival [15,16]. A similar discrepancy has been observed in other tumors such as bile duct cancer and hepatocellular carcinoma [28,39].

Importantly, double positivity for EGFR and HER2, but not single HER2 or EGFR positivity, had a significant correlation with RFS. Previous studies have shown that EGFR and HER2 co-expression was implicated in an increase in tumor aggressiveness and worse prognosis of several cancers [34–36], including cervical squamous cell carcinoma [15]. These results suggest that EGFR and HER2 cooperatively play a crucial role in cancer progression. One possible explanation is heterodimerization between EGFR and HER2. The formation of a heterodimer among RTKs has been shown to modulate signal diversity and signal strength *in vitro* [11,40,41], and it has been shown that HER2 plays a central role in the formation of heterodimers. EGFR preferentially forms heterodimer with HER2 rather than homodimer with EGFR to strengthen broader and longer activation of cellular growth and proliferation signals [15].

Up to date, there are only two reports to examine the expression profile of c-Met in cervical adenocarcinomas including this study. We found that c-Met expression was not correlated with clinicopathological features and prognosis of cervical adenocarcinomas, whereas Tsai et al. demonstrated that its overexpression was correlated with poor prognosis [25]. This discrepancy in results may be due to differences in immunohistochemical and analytical methods.

Our findings indicate a possible therapeutic strategy targeting cell surface RTKs in cervical adenocarcinomas. In particular, EGFR-targeting agents (e.g., cetuximab and panitumumab) or HER2-targeting agents (e.g., trastuzumab) may have efficacy in cervical adenocarcinomas.

In conclusion, RTKs were strongly expressed in cervical adenocarcinoma, and patients who were double positive for EGFR and HER2 showed significantly shorter RFS. Our results suggest that EGFR and HER2 are potential therapeutic targets and that their co-expression of them is a prognostic factor for cervical adenocarcinoma.

## Supporting information

S1 Fig Kaplan-Meier estimates of relapse-free survival (RFS) and overall survival (OS) of patients with cervical adenocarcinoma according to expression of a single RTK. The patients were classified into two groups: those with positive expression of a single RTK (dashed lines) and the remaining patients (solid lines). (a)(b) EGFR, (c)(d) HER2, (e)(f) c-Met.
(PDF)

S2 Fig Kaplan-Meier estimates of relapse-free survival (RFS) and overall survival (OS) of patients with cervical adenocarcinoma according to combined expression of RTKs. The patients were classified into two groups: those with high expression levels of multiple RTKs (dashed lines) and the remaining patients (solid lines). (a)(b) HER2 and c-Met, (c)(d) EGFR and c-Met, (e)(f) EGFR, HER2 and c-Met.
(PDF)
