# Supplementary material for: Prognostic significance of the co-expression of EGFR and HER2 in adenocarcinoma of the uterine cervix
Source: PLoS One. 2017 Aug 31;12(8):e0184123. doi: 10.1371/journal.pone.0184123 (PMC5578660; doi:10.1371/journal.pone.0184123)
Supplement: S1 Fig — The patients were classified into two groups: those with positive expression of a single RTK (dashed lines) and the remaining patients (solid lines). (a)(b) EGFR, (c)(d) HER2, (e)(f) c-Met. (PDF) [file pone.0184123.s001.pdf]

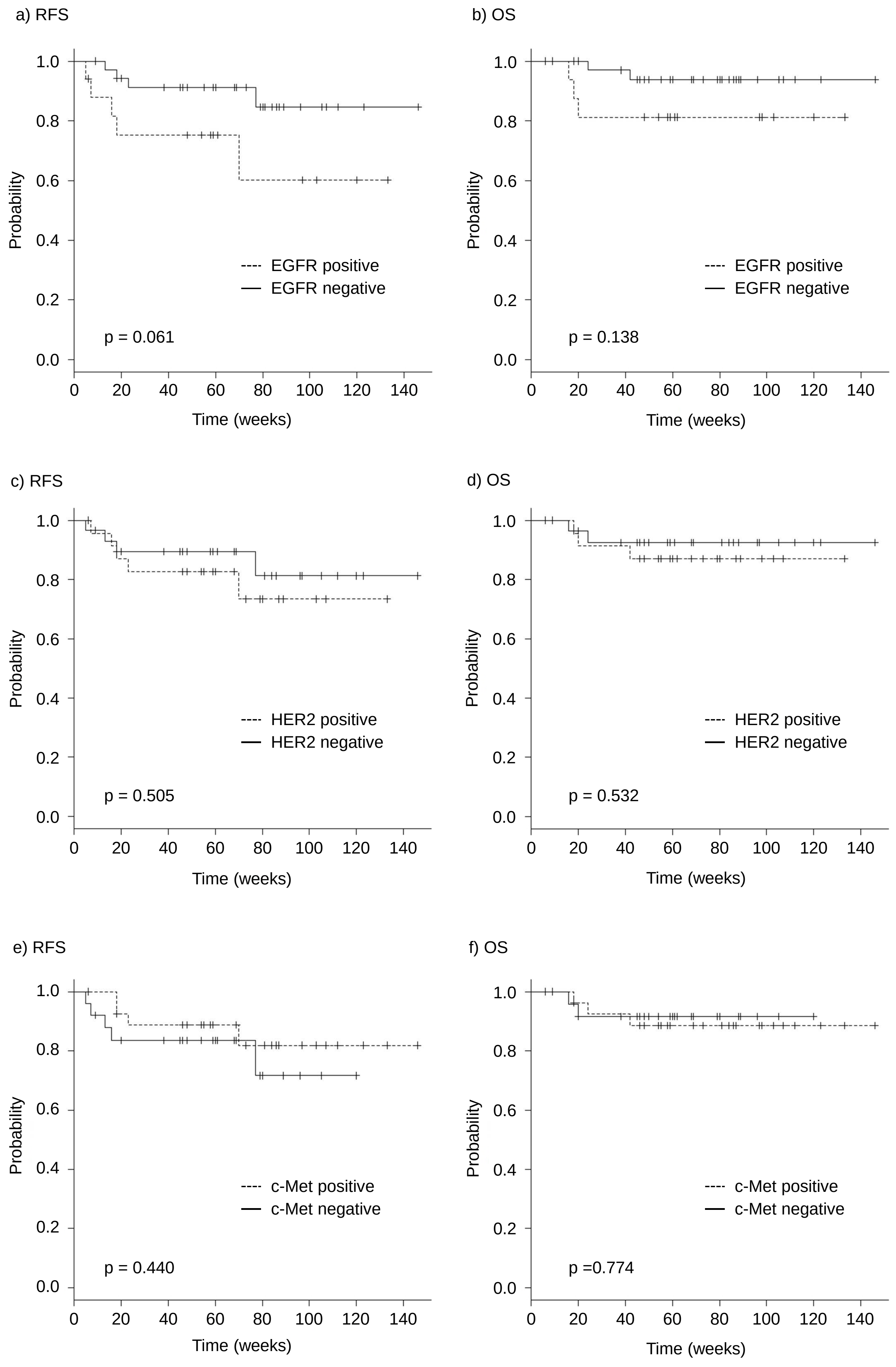

S1 Fig. Kaplan-Meier estimates of the recurrence-free survival (RFS) and overall survival (OS) of the patients with cervical adenocarcinoma according to the expression of single RTK. The patients were classified into two groups, those with high expression of single RTK (dashed lines) and the remaining patients (solid lines). (a)(b) EGFR, (c)(d) HER2, and (e)(f) c-Met.
