# Supplementary material for: Prognostic significance of the co-expression of EGFR and HER2 in adenocarcinoma of the uterine cervix
Source: PLoS One. 2017 Aug 31;12(8):e0184123. doi: 10.1371/journal.pone.0184123 (PMC5578660; doi:10.1371/journal.pone.0184123)
Supplement: S2 Fig — The patients were classified into two groups: those with high expression levels of multiple RTKs (dashed lines) and the remaining patients (solid lines). (a)(b) HER2 and c-Met, (c)(d) EGFR and c-Met, (e)(f) EGFR, HER2 and c-Met. (PDF) [file pone.0184123.s002.pdf]

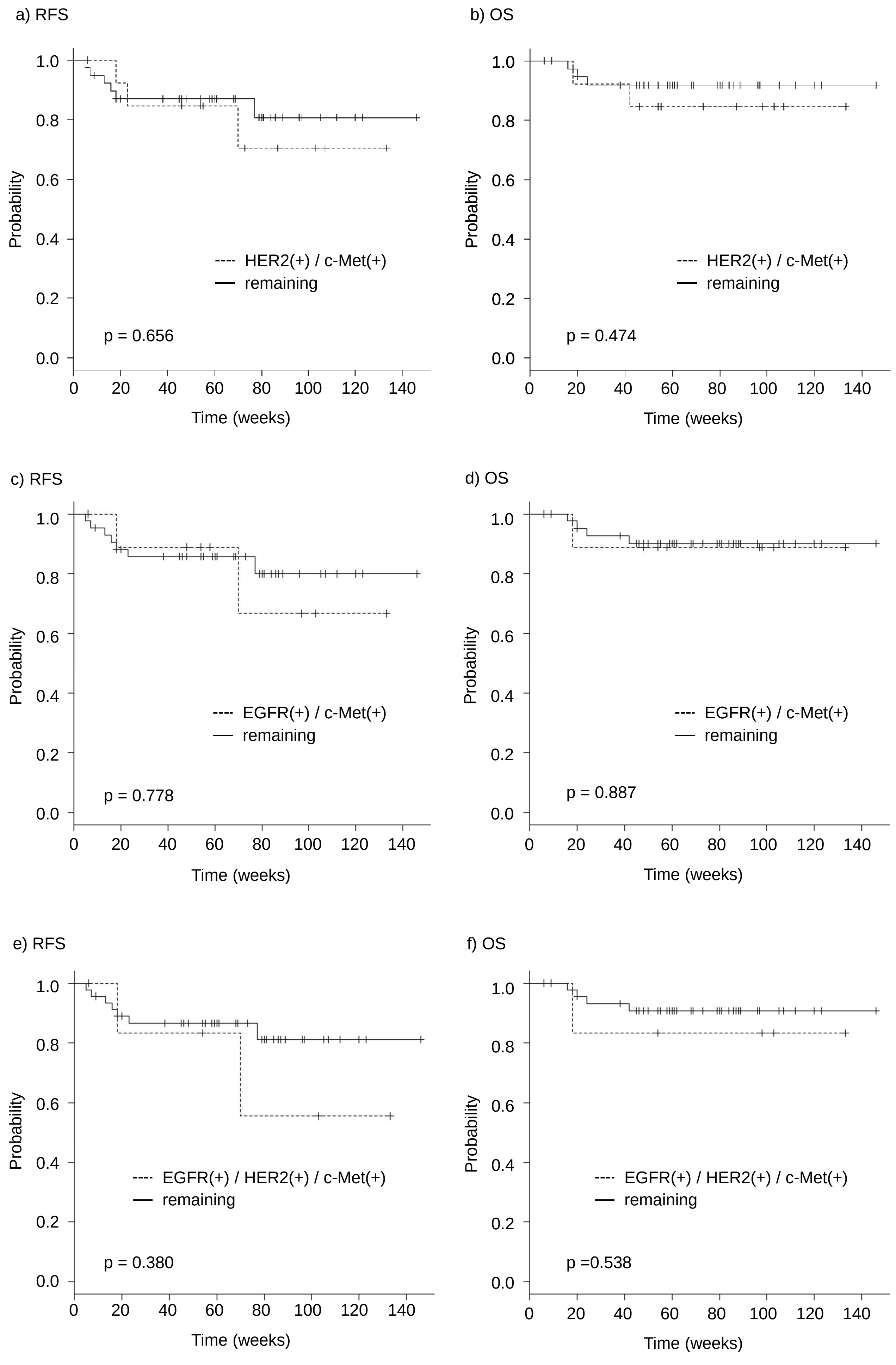

S2 Fig. Kaplan-Meier estimates of the recurrence-free survival (RFS) and overall survival (OS) of the patients with cervical adenocarcinoma according to the combined expression of RTKs. The patients were classified into two groups, those with high expression of multiple RTKs (dashed lines) and the remaining patients (solid lines). (a)(b) HER2 and c-Met, (c)(d) EGFR and c-Met, and (e)(f) EGFR, HER2 and c-Met.
